# Supplementary material for: An immobilized antibody-based affinity grid strategy for on-grid purification of target proteins enables high-resolution cryo-EM
Source: Commun Biol. 2024 Jun 10;7:715. doi: 10.1038/s42003-024-06406-z (PMC11164986; doi:10.1038/s42003-024-06406-z)
Supplement: Supplementary file 2 — Supplementary Information [file 42003_2024_6406_MOESM2_ESM.pdf]

Supplementary Information

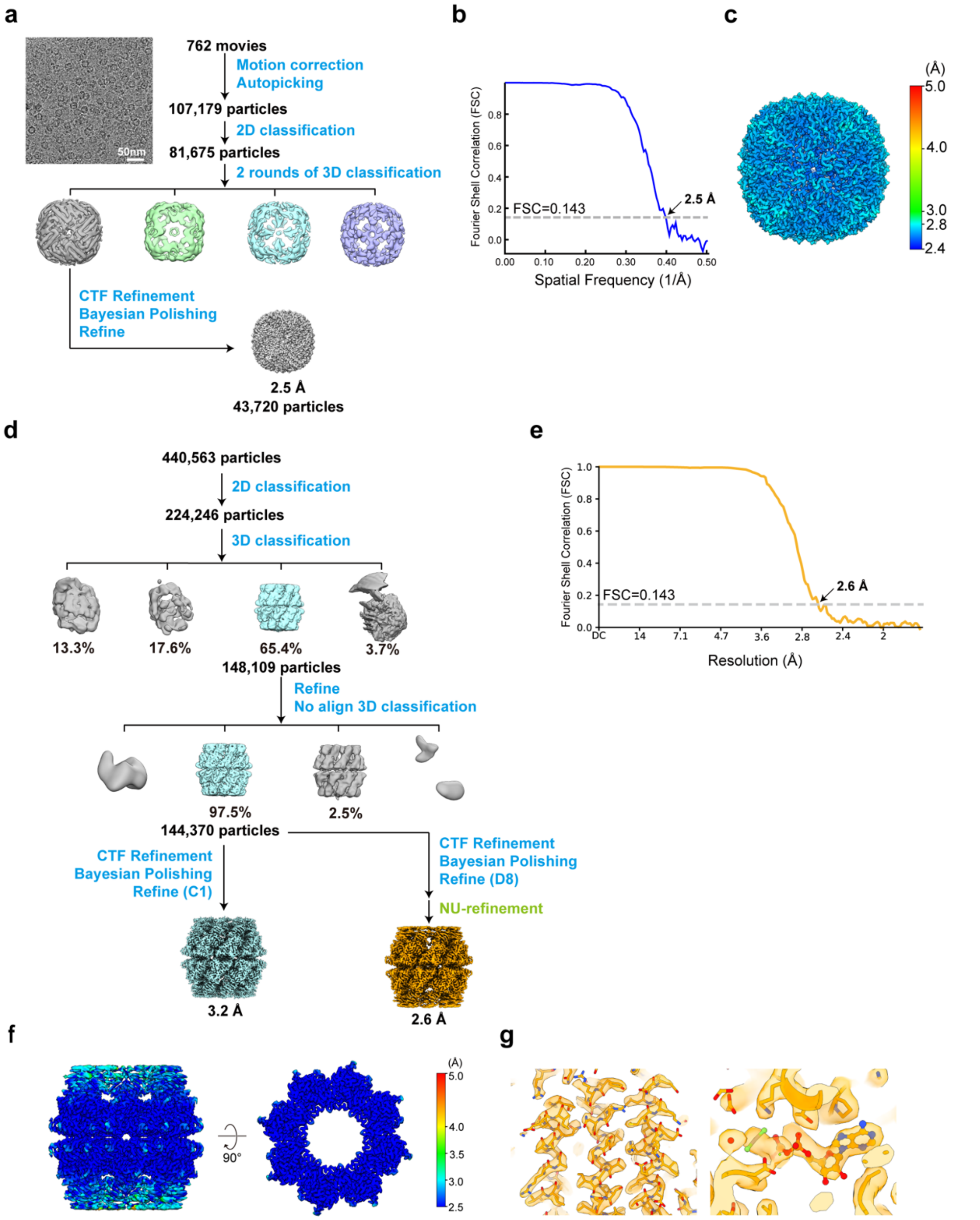

Supplementary Figure 1. Workflow for the data processing of IAAG-treated apoferritin and CCT6-HR-ATP-AlFx. (a) Workflow for the data processing of IAAG-enriched PA-tagged apoferritin. (b-c) FSC curve (b) and local resolution estimation (c) of the PA-tagged apoferritin. (d) Workflow for the data processing of IAAG treated CCT6-HR-ATP-AlFx. The processes labelled in blue were conducted in RELION 3.1 and those in green in cryoSPARC 4.2.1. This color scheme was applied

across all subsequent figures. (e) FSC curve of the CCT6-HR-ATP-AlFx reconstructed with D8 symmetry. (f) Local resolution estimation of the CCT6-HR-ATP-AlFx, displayed are the side view (left) and central slice top view (right). (g) High resolution structure features (left) and the zoom-in view of the nucleotide pocket (right) (model PDB: 5GW5 subunit CCT6).

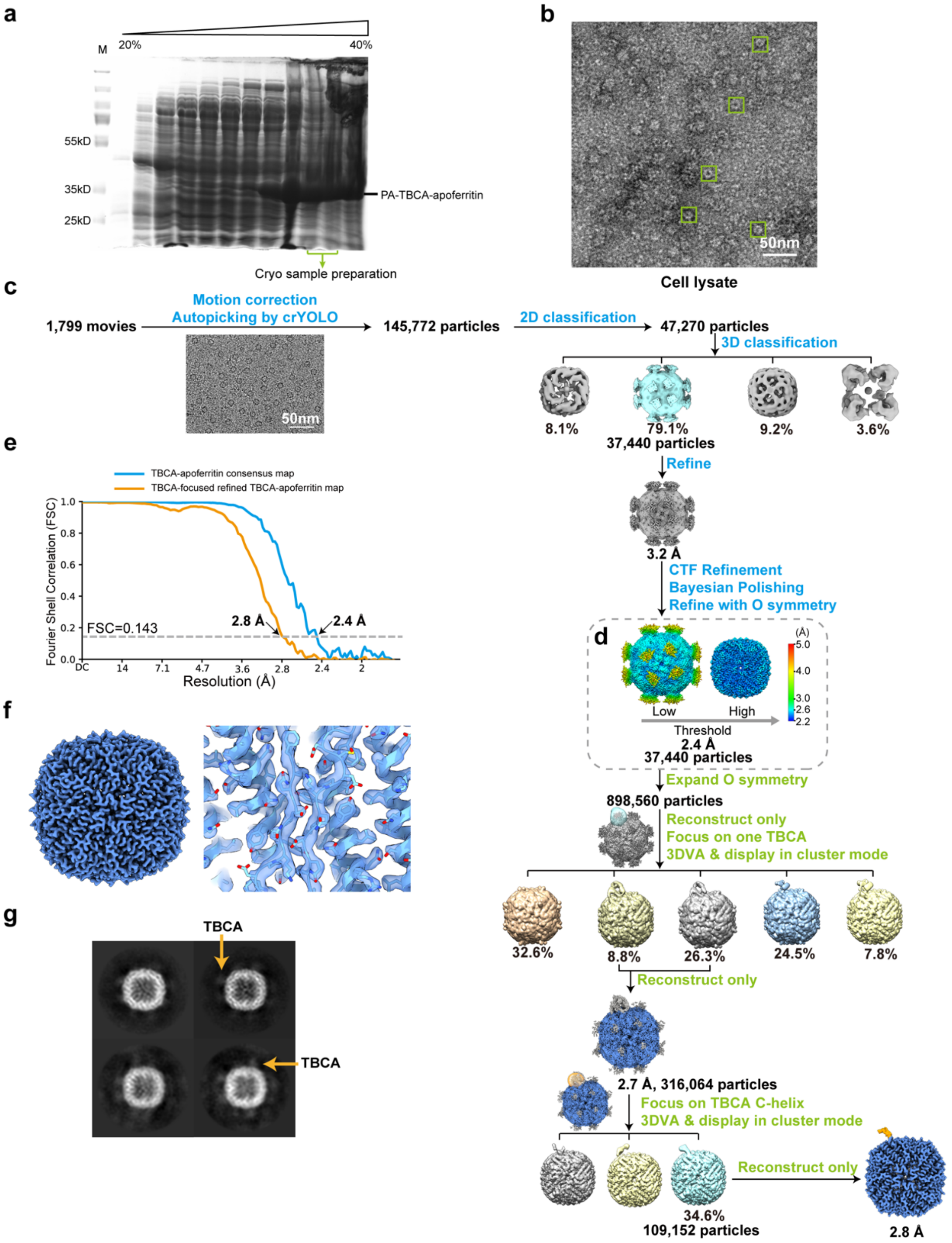

Supplementary Figure 2. Application of the IAAG strategy in the on-grid purification of TBCA-apoferritin from cell lysates and workflow for cryo-EM data processing. (a) Coomassie blue-stained SDS-PAGE of PA-tagged TBCA-apoferritin cell lysates. (b) NS-EM image of TBCA-apoferritin cell lysates, with the TBCA-apoferritin indicated by green square. (c) Workflow for data processing of the IAAG

on-grid purified TBCA-apoferritin. (d) The local resolution map of the 2.4-Å-resolution consensus map of TBCA-apoferritin. At lower rendering threshold (left), the 24 displayed TBCAs become visible, while at higher rendering thresholds (right), the high-resolution structure features of the apoferritin scaffold are clearly discernible. (e) FSC curve of the TBCA-apoferritin maps. (f) The high-resolution structure features of the 2.4-Å-resolution consensus map of the on-grid purified TBCA-apoferritin, showing the well-resolved apoferritin portion (PDB: 6Z6U). (g) 2D class averages of TBCA-apoferritin, which displays fuzzy TBCA density (indicated by orange arrow) around apoferritin.

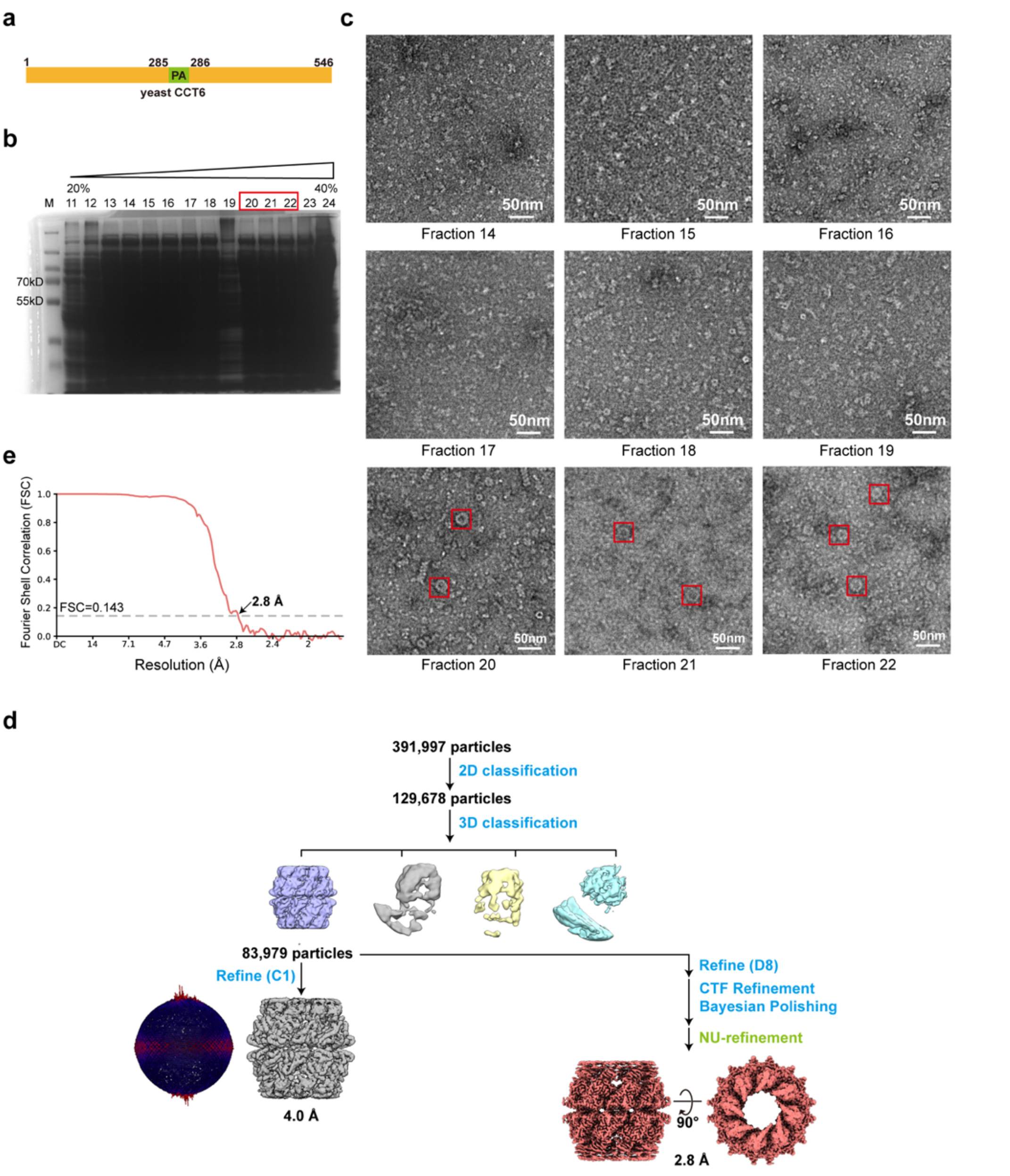

Supplementary Figure 3. Workflow for data processing of the IAAG on-grid purified CCT6-HR. (a) Diagram of the PA tag inserted yeast CCT6 plasmid construct. (b) Coomassie blue stained SDS-PAGE of CCT6-HR cell lysates. (c) NS-EM images of CCT6-HR crude cell lysate fractions, with most of the fractions showing no observable target. Only in fractions 20-22, very sparsely distributed ring-shaped particles (indicated by red squire, likely present CCT6-HR) can be seen. (d) Workflow for the data processing of the IAAG on-grid purified CCT6-HR. Euler angle distribution for the C1 reconstruction is also showed. (e) Resolution assessment by the FSC curve of the CCT6-HR map.

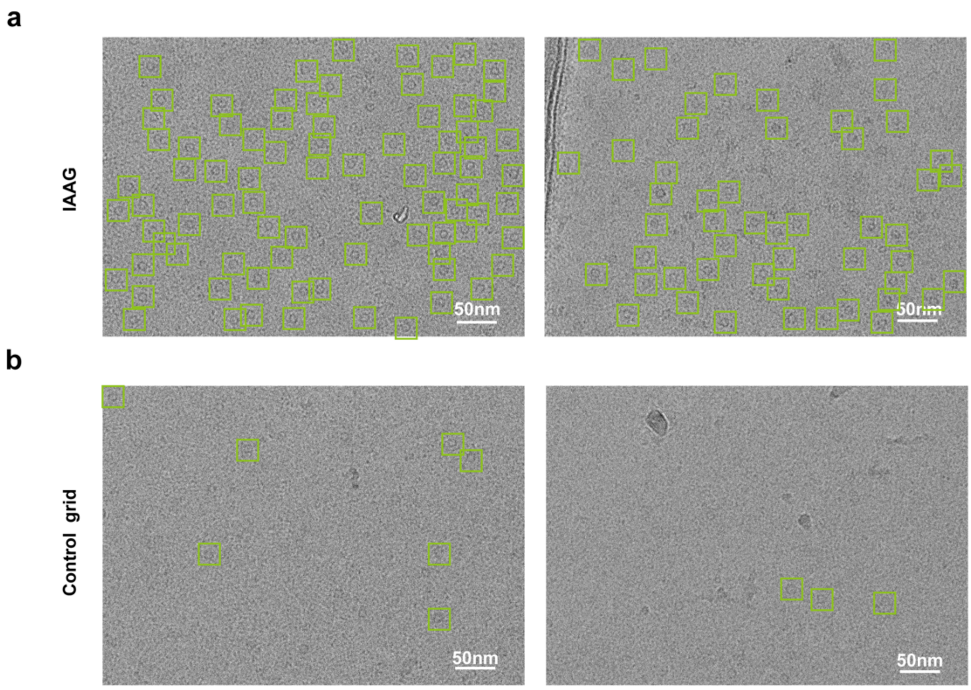

Supplementary Figure 4. On-grid purification of TBCA-apoferritin using IAAG strategy, assembled with continuous carbon-covered grid. (a) Representative cryo-EM images of PA-tagged TBCA-apoferritin, on-grid purified from cell lysates using IAAG grid supported by continuous carbon film. TBCA-apoferritin are indicated with green squares. (b) Representative cryo-EM images of the control continuous carbon grid treated with cell lysates.

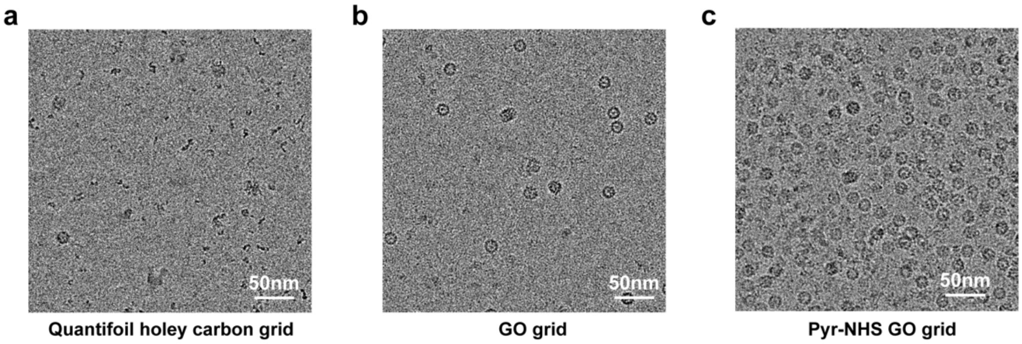

Supplementary Figure 5. Application of Pyr-NHS coated grid for immobilization and enrichment of purified proteins. (a-b) Representative cryo-EM image of the CCT6-HR-ATP-AlFx prepared using a Quantifoil holey carbon grid (a) or a GO grid (b). (c) Cryo-EM image of the CCT6-HR-ATP-AlFx using a Pyr-NHS coated GO grid, illustrating a great enrichment of particles using this type of grid. These observations demonstrate the effeteness of Pyr-NHS coated GO grids in immobilizing and enriching purified proteins for cryo-EM studies.

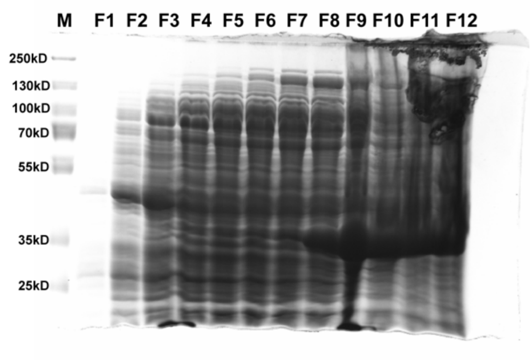

Supplementary Figure 6. Uncropped image related to Supplementary Figure 2a.

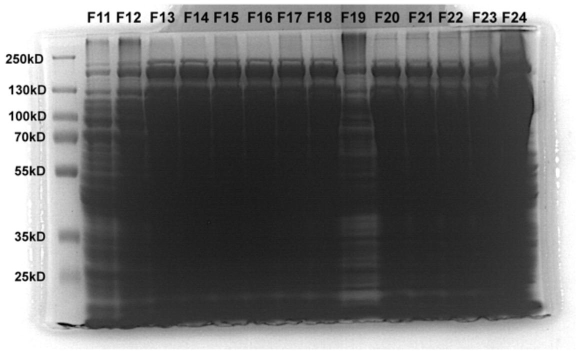

Supplementary Figure 7. Uncropped image related to Supplementary Figure 3b.
