# Supplementary material for: An immobilized antibody-based affinity grid strategy for on-grid purification of target proteins enables high-resolution cryo-EM
Source: Commun Biol. 2024 Jun 10;7:715. doi: 10.1038/s42003-024-06406-z (PMC11164986; doi:10.1038/s42003-024-06406-z)
Supplement: Supplementary file 3 — Description of Additional Supplementary Files [file 42003_2024_6406_MOESM3_ESM.pdf]

Description of Additional Supplementary Files

File name: Supplementary Movie 1

Description: Cryo-ET analysis of the IAAG ongrid purified TBCA-apoferritin sample. The slices are displayed from the air-water interface to beyond the GO surface. The PA tagged TBCA-apoferritin distributed near the GO grid surface and away from the AWI.
